# Letter on *Predicting the number of sites needed to deliver a multicentre clinical trial within a limited time frame in the UK*

**DOI:** 10.1186/s13063-020-04798-x

**Published:** 2020-10-21

**Authors:** Rosemary Greenwood, Julie Pell, Paula Foscarini-Craggs, Katharine Wale, Ian Thomas, Charlotte Bradbury

**Affiliations:** 1 School of Translational Health Sciences, University of Bristol, Bristol, UK; 2 University Hospitals Bristol and Weston NHS Foundation Trust, Bristol, UK; 3 Cardiff University Centre for Trials Research, Cardiff, UK

**Keywords:** Multicentre randomised controlled trials

## Abstract

**Abstract:**

When planning a multicentre clinical trial, it can be difficult to predict the time needed to open individual sites, and this in turn impacts on the total number of sites needed, the budget and the time frame for a clinical trial to be delivered successfully. This is of particular importance for funding applications with a limited time frame and budget such as NIHR RfPB. It is more efficient and cost-effective to open the total number of sites needed at the outset of a trial, rather than to respond later to slow site opening and recruitment. Here, we share our experience of successfully delivering a multicentre clinical trial for a rare disease within a limited time frame and budget by approximately doubling the number of sites initially predicted to be needed. We initially predicted 20 sites would be needed to deliver the clinical trial, but early on in the trial, the number of sites was more than doubled to allow successful recruitment of the target sample size within the desired time frame. Of the 48 ethically approved sites, the median time from ethical approval of a site to opening for recruitment was 182 days (95% confidence interval [143 to 245 days]) and ranged from 18 to 613 days. In four (9%) of these sites, part of the delay was due to pharmacy sign off not being given when R&D had issued capacity and capability (C&C). Delays due to pharmacy sign off varied from 10 days to over 3 months delay in two sites (94 days and 102 days). A mathematical solution to the problem of planning a study with a short recruitment window has been given to support the planning and costing of grants with fixed time constraints: number of sites = *required sample size divided by (number of eligible patients per site per month times recruitment rate times (the number of months accrual minus 6 months)).* We expect these results to help others who are planning multicentre clinical trials in the UK. Ethical approval from NRES Committee South West (IRAS number 225959).

**Trial registration:**

EudraCT Number 2017-001171-23. Registered on 26 June 2017

## Background

Grant applications made to the NIHR Research for Patient Benefit (RfPB) funding stream may be for a maximum total duration of 3 years from start to finish (https://www.nihr.ac.uk/explore-nihr/funding-programmes/research-for-patient-benefit.htm). Allowing for 3 months study set-up, 12 months patient follow-up, 3 months data cleaning and analysis and 3 months write up, this leaves only 15 months for site set-up and patient recruitment. Although grants often estimate 3 months to set up and open sites in the UK, it has been unclear whether this is realistic due to the number of processes required. In the UK, a central study set up includes ethics and regulatory approval, study documents including protocol, sponsor and database set up as well as formation of trial management groups. Site set up and opening requirements include capacity and capability sign off, pharmacy approval, staff delegation logs and site initiation visits.

We successfully applied for NIHR RfPB funding for a multicentre-randomised controlled trial in patients with immune thrombocytopenia (ITP) [1]. As this is a rare disease (incidence 2–3/100,000), multiple sites were needed to recruit sufficient patient numbers to reach a statistically valid sample size (*n* = 120). The grant application included an initial estimation that 20 sites would be needed to achieve this sample size, allowing 3 months to set up and open these sites and 15 months to recruit patients. Here, we demonstrate why the actual number of sites needed was far greater than anticipated due to the longer time frame needed to open sites, which was highly variable and rarely within 3 months. Early on in the trial, the number of sites was more than doubled to allow successful recruitment of the target sample size within the desired time frame. These results may help others when planning the time frame and number of sites needed for a multicentre clinical trial in the UK.

## Methods

Following ethical approval, all sites were sent the trial paperwork and capacity and capability sign off was requested. In addition, pharmacy sign off and delegation logs were sought. The site initiation visits were performed remotely, enabling flexible and timely availability from the trial team. The sponsor issued permission to the co-ordinating trials unit to proceed with opening recruitment at each site, once it was satisfied that all the necessary study-wide actions had been undertaken prior to the first site opening to recruitment. Site recruitment was monitored by the overseeing trials unit. Each month, sites provided screening reports, and all Trial Management Groups were updated on screening and recruitment numbers, with comparison to projected target recruitment. Non-recruiting sites were contacted personally by the chief investigator, and regular newsletters were sent to sites with information about recruitment rates.

The time it took from formal ethical approval of participating sites to being fully open to recruitment has been plotted using a Kaplan-Meier curve. The Kaplan-Meier was used to produce the median delay with 95% confidence intervals. We also looked at the time to the recruitment of the first patient for all sites that were open to recruitment. In each case, sites that did not open before the study closed or sites which did not recruit before the study closed had their data censored at that point.

For some sites, local confirmation of C&C and the green light from the sponsor was given prior to pharmacy sign off and the proportion of sites delayed by this, and the time delay this produced has also been summarised.

A mathematical solution to the problem of planning a study with a short recruitment window has been given to support the planning and costing of grants with fixed time constraints. The formula is given in the last paragraph of the “Creation of a method for predicting recruitment requirements” section.

## Results

At the stage of funding application, we had estimated that 20 sites would be needed to achieve a recruitment of 120 patients within 15 months. Initially, ethical approval was sought for 29 sites to allow recruitment contingency, and we received ethical permission from these hospitals to be participating sites on 12 June 2017. Following this, to boost recruitment further, we added another two sites in September 2017, 10 November 2017, 6 January 2018 and the final one in June 2018.

Of the total 48 sites included in the ethics submissions, 43 (90%) opened to recruitment, one of which was closed to recruitment a month later due to a change in PI who declined to sign the delegation log. The remaining five sites failed to open for a variety of reasons: PI change, lack of staff/trial team/pharmacy capacity, restructuring and no further contact. The additional sites were set up within the initially funded grant and without extra cost to the funder. The cost of the site set up was minimised by remote site initiation visits, and within the grant, hospitals received a payment for each patient recruited. In addition, in the UK, sites receive some financial support to set up NIHR portfolio studies like this.

**Fig. 1:**
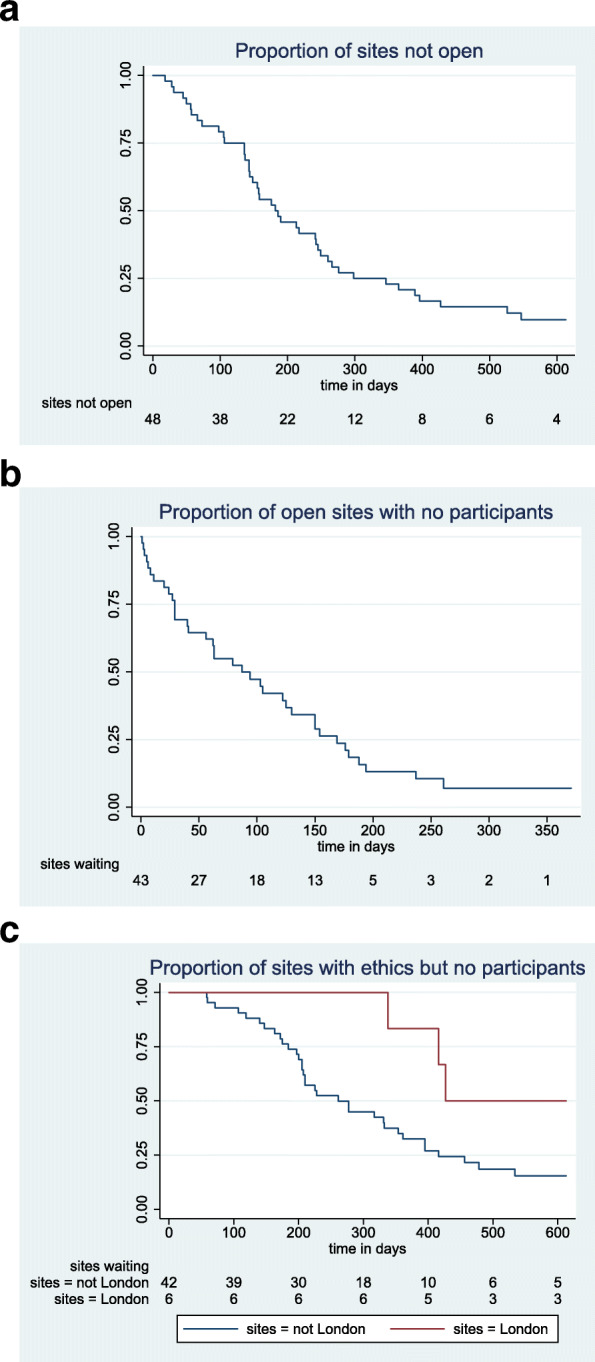
**a** Proportion of sites not open. **b** Proportion of open sites with no participants. **c** Proportion of sites with ethics but no participants

The median time from ethical approval of a site to being open to recruitment was 182 days (95% confidence interval [143 to 245 days]) and ranged from 18 to 613 days for one site that opened very close to the end date and did not recruit any patients. This is represented in the Kaplan-Meier curve in Fig. 1a.

Part of the delay for 4 (9%) sites was due to pharmacy sign off not having been given before the site issued C&C. Delays due to pharmacy sign off varied from 10 days delay to over 3 months delay in two sites (94 days and 102 days).

In addition, further paperwork was requested for seven trusts (16%) at which the PI changed or changed their name—one of which did not re-open to recruitment as mentioned earlier.

Of the 43 open sites, 37 recruited to the study. The median time from opening to the recruitment of the first patient was 87 months [95% CI 41 to 130 days]. Only 13 of the 34 sites (35%) were able to recruit the first patient within 30 days of opening which was expected due to the rare nature of this disease.

Compared to the rest of the UK, the six sites based in London were slower to open (median of 242 days) and to recruit the first patient (median of 150 days) although this did not reach statistical significance (*p* values of 0.21 and 0.07, respectively, using the log rank test). When these two delays were combined, the resulting total time to the recruitment of the first patient at London sites had a median time of 427 days compared with non-London sites of 261 days (*p* = 0.045) as seen in Fig. 1c.

### Creation of a method for predicting recruitment requirements

A predicted recruitment from 20 sites with a 15-month recruitment window and an average of 3-month delay would have produced 240 site months. With a median of 5.2 months delay, rising to 6 months when pharmacy sign off was included, the number of site months these 20 could produce is more likely to be 20 × (15–6) = 180 which is 75% of the original prediction.

To compensate for an unexpectedly slow set-up, additional sites are usually added in a later ethics amendment. Setting these sites up later means that their available recruitment window is shortened by both a 6-month latency in recognising the need to increase the number of sites and a 6-month latency in time to being open to recruitment. Therefore, to make up the 25% lost capacity in a 15-month recruitment window (a difference between the required 240 site months and the predicted 180 site months giving a short fall of 60 site months), a study would need to recruit an additional 50% more sites (60/(15 − 6 − 6) = 20 sites).

The most efficient method is to open additional sites from the start of the trial. Not only does this increase the potential recruitment window at sites, but it also reduces the trial team resource needed to submit additional ethical amendments and later site initiation visits. A study that needed 20 sites for 12 months would recruit 27 sites for a 15-month accrual period with the knowledge that some sites would be much slower than others to open for recruiting with an average latency of 6 months. If the accrual period is limited to 12 months, the number of sites that the study would need to involve doubles to 40.

The formula for the number of sites required that could be used to plan a future trial becomes: *required sample size divided by (number of eligible patients per site per month times recruitment rate times (the number of months accrual minus 6 months)).*

## Conclusions

Large multisite clinical trials of a medicinal product can pose particular challenges when costing the grant application, owing to the significant variation in the length of time it takes sites to open to recruitment. A trial with a short recruitment window will suffer the most from delays in this process. For example, to complete a trial with a 12-month recruitment window to time and target, double the number of sites may be needed. Funders may need to be aware of the increased costs associated with the burden of obtaining additional sites or might consider allowing extensions for trials of rare diseases. In addition, close monitoring of site opening and recruitment are also essential, with proactive measures taken if the trial is behind target.

## Data Availability

Not applicable
